# A literature review on dental autopsy – an invaluable investigative technique in forensics

**DOI:** 10.4322/acr.2021.295

**Published:** 2021-08-20

**Authors:** Jyotirmoy Roy, Ujwal Shahu, Payal Shirpure, Supriya Soni, Utsav Parekh, Abraham Johnson

**Affiliations:** 1 National Forensic Sciences University, School of Forensic Science, Laboratory of Forensic Odontology, Gandhinagar, Gujarat, India; 2 Pramukhswami Medical College, Department of Forensic Medicine and Toxicology, Gujarat, India

**Keywords:** Postmortem, Autopsy, Surgical Incision, Osteotomy, Forensic Dentistry

## Abstract

Forensic odontology is a specialty of dental sciences that deals with dental evidence in the interest of the justice system. The science of autopsy has been developing from the ancient times even before the popularization of general medicine. The objective of a medico-legal autopsy is to identify significant clues for an ongoing forensic investigation. However, in certain circumstances, it is difficult to conduct an oral examination owing to the anatomic location of the oral cavity. The onset of rigor mortis after death poses further complications. Thus, skillful and sequential dissections of the oral and para-oral structures are required to expose the dentition. Dental autopsy includes incisions and resection of the jaw for the detailed examination of the oral cavity. The procedure involves various modes of examination, including visual and radiographic, which help in human identification in forensic investigation. The present paper provides an overview of the various methods of dental autopsy.

## INTRODUCTION

The meaning of autopsy is “to see for oneself,” and in the modern context, it denotes the postmortem examination of the dead.^1^ There are three types of autopsies - anatomical autopsy for educating the medical and para-medical students, pathological autopsy for evaluating the extent of disease after the death of the patient, and medico-legal autopsy performed to aid the justice system.^2^ The first-ever human cadaver dissection for academic purpose was done in Greece in 3^rd^ century BC.^3^^,^ 4 The first-ever medico-legal autopsy was conducted in mid-13^th^ century at the University of Bologna in Italy.^5^ Bartolomeo da Varignana is thought to have performed the first autopsy in a case of poisoning in 1302.^6^ The fundamentals of modern autopsy were introduced by Friedrich Albert Zenker (1825–1898) and Rudolf Virchow (1821–1902) in 19^th^ century in Germany.^7^^,^ 8 Each medico-legal autopsy includes a thorough external examination of the body, followed by a complete exploration of all the body cavities. During the external examination, multiple metric and non-metric data such as body weight, body length, chest circumference, general built, and nutrition level are fetched.^9^ The internal examination includes dissection of all the body cavities to discern the cause, manner, and time of death.

Forensic odontology is a specialized branch of dentistry that deals with the proper handling and inspection of dental evidence as well as the systematic evaluation and presentation of dental findings.^10^ This field dates back to 49 AD in the Roman Empire, where teeth played a vital role in identification.^11^ Human teeth are considered as primary identifiers for an individual. Even the “INTERPOL” protocols for Disaster Victim Identification clearly state that a dentist with medico-legal training should examine the teeth and jaw whenever appropriate.^12^ Dental autopsy refers to the elaborate investigation of the oral and para-oral structures for legal purposes.

A dental examination is performed in the absence of rigor mortis (stiffening of the masticatory muscles), especially immediately after death, or in the advanced stages. If rigor mortis sets in, exposure of the oral cavity becomes very difficult.^13^ In such cases, dental profiling is achieved by making a systematic incision and reflection of the skin, muscle, and fascia for a proper view of the oral cavity. This step is important for the identification of the deceased as it allows radiographic, photographic, and visual examination of the oral cavity.^14^ It is recommended in specific conditions such as advanced decomposition, charred victims, and traumatic death involving ballistic or weapon injury.^15^

## METHODS OF DENTAL AUTOPSY

Dental autopsy encompasses a thorough external examination of the oral structures to identify injuries, froth/liquid, or foreign objects. The internal examination of the oral cavity requires specific dissection techniques that provide a full view of the inner structures. Two approaches are available to view the dental structures, namely the incision method and the jaw resection method. The incision method incorporates various incisions to reflect the skin, muscle, and fascia. Subsequently, the dentition is exposed while preserving the anatomic relationship of the two jaws.^16^ In the resection method, the maxilla and mandible are entirely resected and removed separately.^17^

### Incision Method

**Figure 1:**
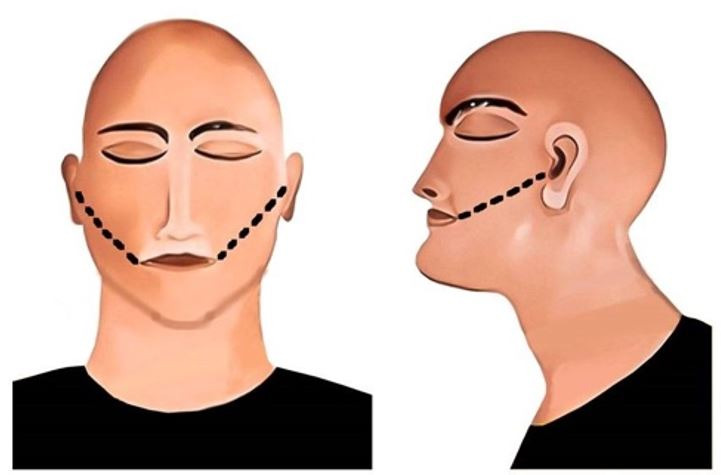
Luntz-Luntz incision method; black line represents the incision. A – frontal view; B – lateral view.

The first-ever incision on the face for exploring the oral cavity was described by Luntz and Luntz in 1974. A “V-shaped” incision extending from the labial commissure of the mouth to the auricular region was made to visualize the vestibular and buccal surfaces of the teeth (Figure 1).^18^ With the increasing need of dental autopsy, numerous incision methods have been introduced.

#### Extra-Oral Incision

**Figure 2:**
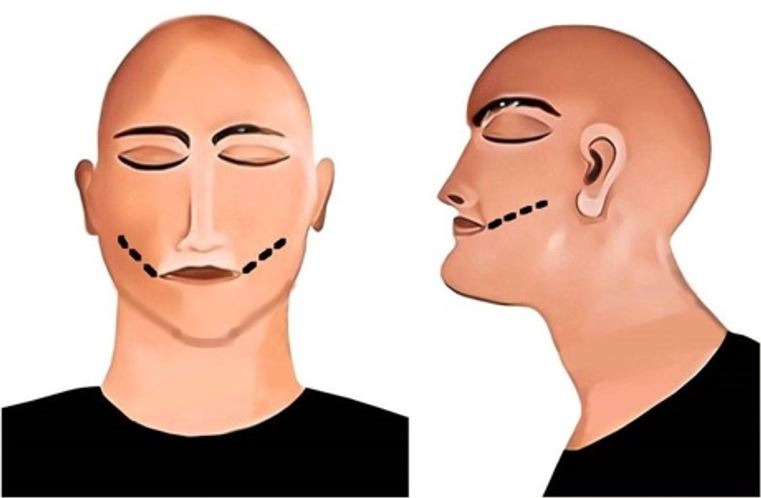
Extraoral incision method; black line represents the incision. A – frontal view; B – lateral view.

Bilateral incisions are made from the oral commissures to the body of the ramus on a plane parallel to the plane of occlusion. The incision runs full depth through the skin, fat, fascia, and the masseter muscle, reaching the lateral aspect of the ramus and the buccal aspect of the teeth. Dissection of the masseter muscle is often sufficient to expose the oral cavity, but traction instruments are required at times to pry open the mouth (Figure 2).^19^

#### Infra-Mandibular Incision

**Figure 3:**
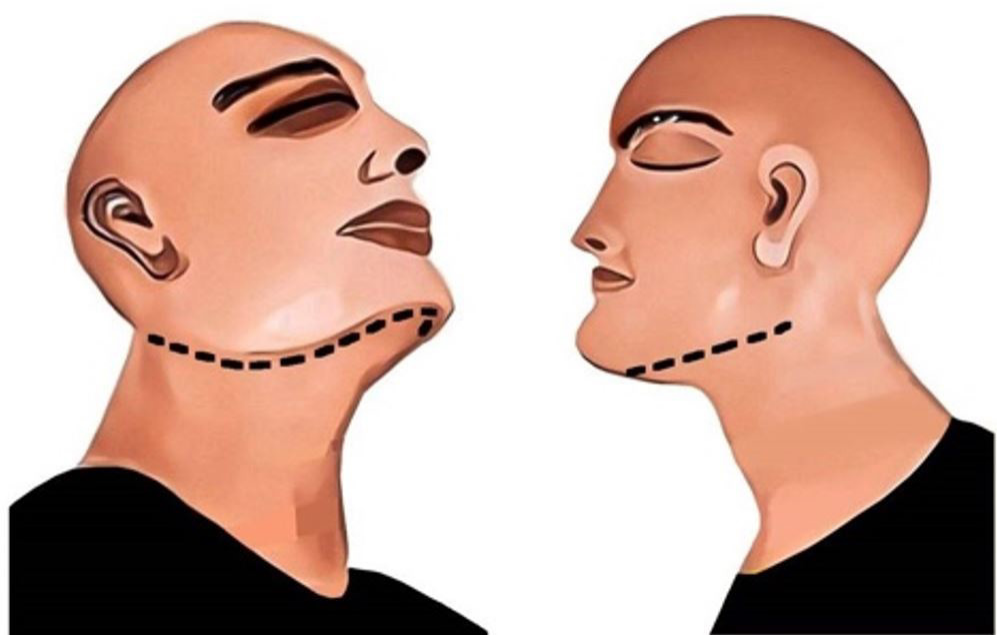
Inframandibular incision method; black line represents the incision. A – oblique lateral view; B – lateral view.

An incision is made through the full length of the skin, fat, and muscles from the angle of the mandible to the midline of the chin. The incision follows the inferior border of the mandible to allow soft tissue dissection in an upward direction, further dissecting the attachment of the masseter muscle from the bone and the vestibular musculature. The presence of rigor mortis may necessitate the use of traction instruments to pry open the mouth. The retromolar pad of the mandible can also be lowered to increase visibility (Figure 3).^20^

#### Aka–Canturk Method

**Figure 4:**
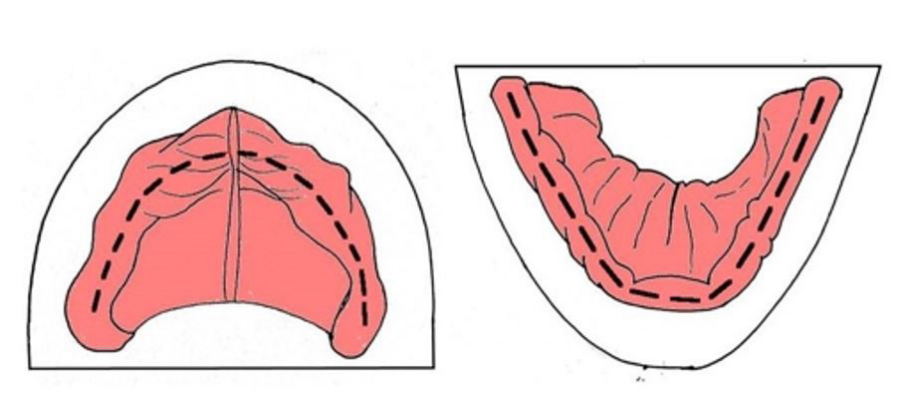
Aka–Canturk incision method; black line represents the incision. A – maxillary arch; B – mandibular arch.

This method involves the excision of the tooth germ of a neonatal subject for accurate age determination. The oral cavity is pried open, and an incision is made along the curvature of the edentulous dentition. The oral mucosa is then reflected by dissecting the attached gingivae. Next, the periosteum is removed to expose the alveolar bone, allowing collection of the tooth germ (Figure 4).^21^

#### Oropharyngeal Complex Dissection

In this method, the soft tissue of the neck is dissected to obtain a good view of the dentition and its adjacent structures. The tip of the tongue is pushed upward and backward with forceps. A knife is inserted under the chin through the floor of the mouth, and an incision is made along the sides of the mandible to reach the angle of the mandible, where the blade is turned inward to avoid dissection of the carotid artery. The tongue is pushed down under the mandibular arch. Subsequently, the soft palate is cut to include the uvula and tonsils with the tongue and the neck organs. At this stage, the knife is carried backward and laterally on both sides of the midline dividing the posterior pharyngeal wall. The larynx is retracted to dissect the pharyngeal tissues from the posterior, anterior, and lateral aspects. The pharynx is then pulled down to the upper part of the neck. The dissection is then continued distally through the pre-vertebral muscles in the anterior surface of the cervical vertebrae. This sequence completes the dissection of the neck structures up to the level of the suprasternal notch, and the organs are removed en-bloc.^22^

#### Silver and Souviron Incision

**Figure 5:**
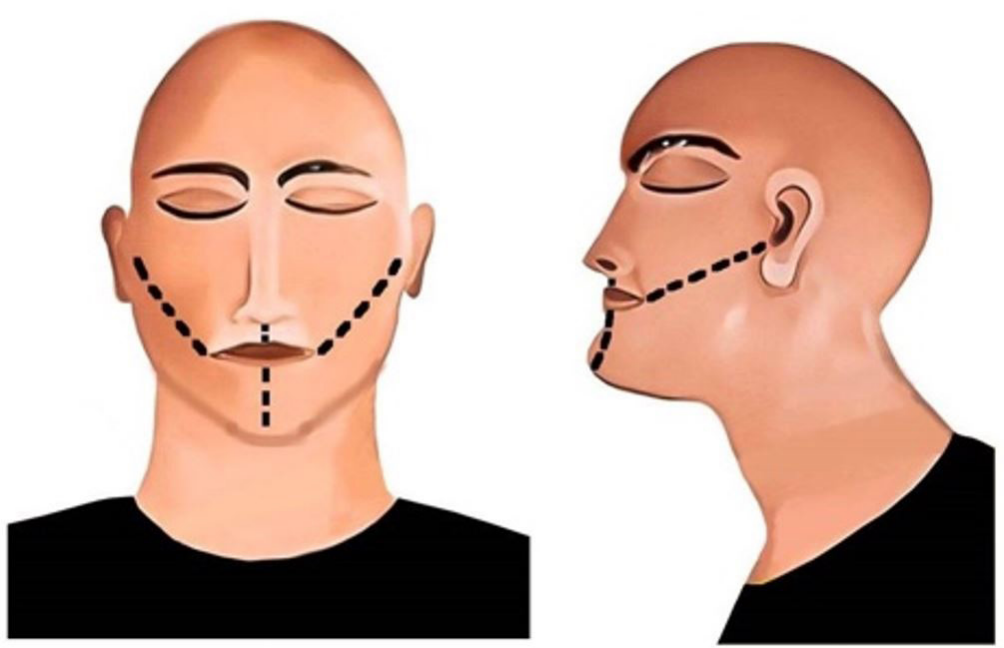
Silver and Souviron incision method; black line represents the incision. A – frontal view; B – lateral view.

A horizontal incision is made originating from the commissure of the lip and terminating in the tragus of the ear. The incision is made bilaterally, running through the full depth of the skin, fat, and muscle layers. A vertical incision is made along the midline of the upper and lower lips. For the upper lip, the incision terminates at the root of the nasal bridge, and for the lower lip, it terminates at a level enabling sufficient tissue retraction. The soft tissue is then retracted with the help of an upward blunt dissection in case of the upper lip and downward blunt dissection in case of the lower lip. Once the soft tissue flaps are adequately retracted, the teeth can be visualized (Figure 5).^23^

#### “C” Shaped Incision

**Figure 6:**
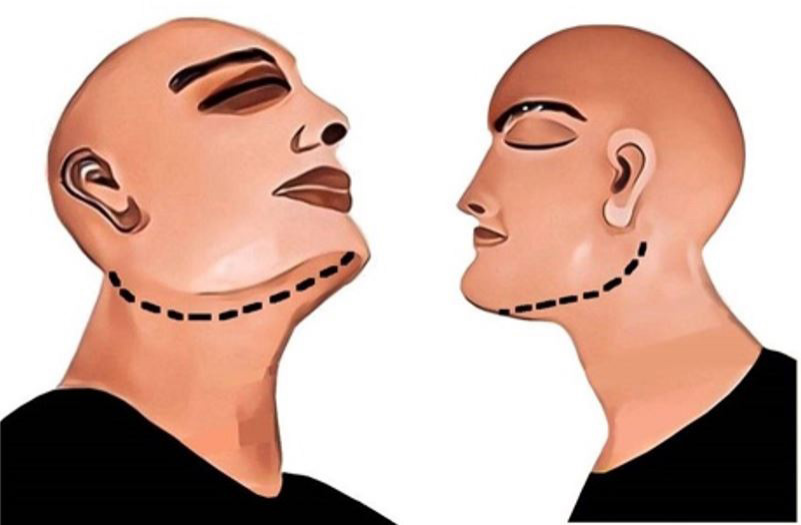
“C” Shaped incision method; black line represents the incision. A – oblique lateral view; B – lateral view.

Bilateral “C-shaped” incisions are made in the retromandibular region. The upper limit of each incision lies 1 cm below the ear lobe, and the lower limit is extended to a virtual point on the inferior border of the mandibular body. The incision is made in such a way that it follows the posterior border of the mandible at a minimum distance of 2 cm. The length of the whole incision is about 6 cm. The incision should be deep enough to cut the skin, fat, fascia, and muscle. The deep layers of the medial pterygoid and masseter muscles and the respective tendon insertions are dissected around the mandibular angle. Linear osteotomy is performed to completely separate the ramus from the body of the mandible. As the major incision lies behind the ear, it is aesthetic and preserves the anatomic continuity of the face (Figure 6).^24^

#### Fereira et al. Method

**Figure 7:**
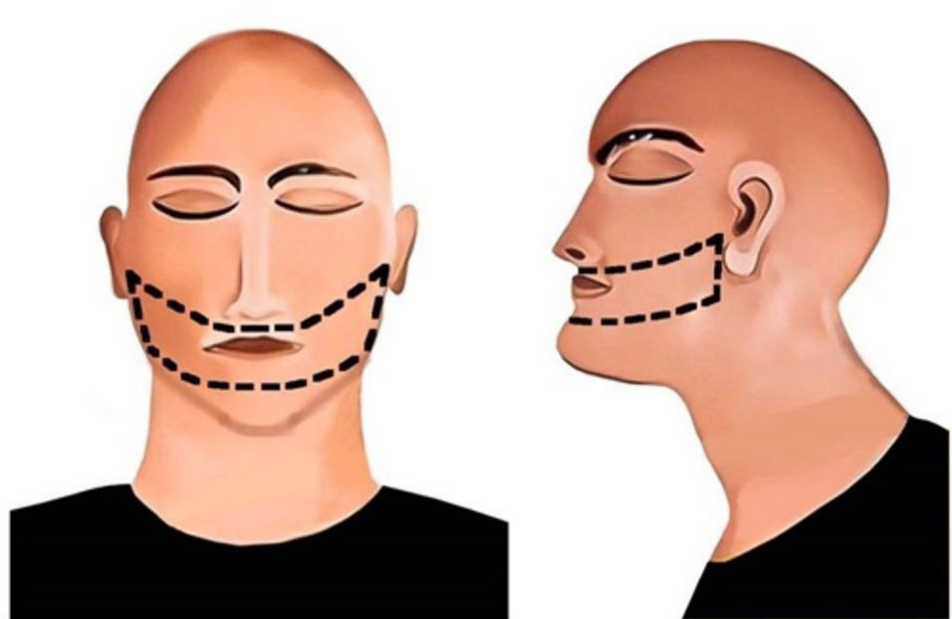
Fereira et al. incision method; black line represents the incision. A – frontal view; B – lateral view.

In this method, the skin, fat, and muscle overlying the dentition are removed in such a way that postmortem approximation or reconstruction is easy and has an aesthetic appeal. In this technique, three types of incisions are made in the mid-face region, namely superior, inferior, and lateral incisions (Figure 7).^25^

- Superior incision – An incision is placed on one side of the tragus and is continued to the opposite side through the anterior nasal spine. The incision should go full depth, and the blade should contact the bony surface.^25^
- Inferior incision – The incision is placed on the mental eminence of the jaw, and is continued bilaterally through the base of the alveolar process sideways to the body of the mandible, parallel to its inferior edge. The incision should cover the width of the ramus, terminating at its posterior border and subsequently dissecting the masseter muscle.^25^
- Lateral incisions – Two vertical incisions are placed, joining the distal ends of the superior and inferior incisions. The lateral incisions act as relief incisions, allowing en bloc removal of the tissues overlying the dentition.^25^

After careful removal of the lip and cheek tissues, the internal pterygoid muscles are dissected. The condyle and capsular ligaments of the temporo-mandibular joints are dissected. Once all the attachments are removed, the mandible is disarticulated.

#### Nossintchouk et al. Method

**Figure 8:**
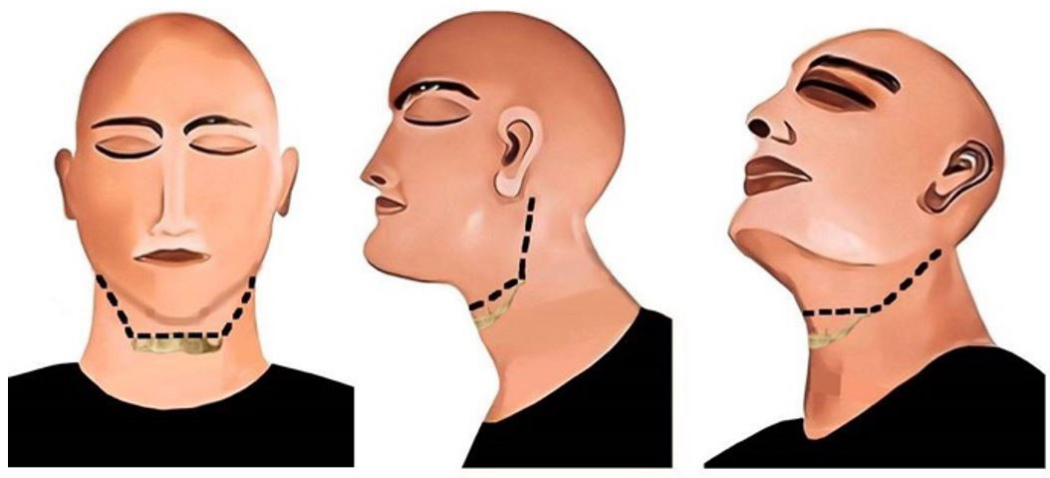
Nossintchouk et al. incision method; black line represents the incision. A – frontal view; B – lateral view; C – oblique lateral view.

In this method, an incision is made from the level of the hyoid bone and is extended in an upward and backward direction. The incision is continued till the back of the ear is reached, and is terminated there to provide good tissue relief. It should run through the full length of the skin, fat, fascia, and muscles. Furthermore, it should be performed bilaterally to appear as a “V,” with its apex at the level of the hyoid bone (Figure 8). Next, the flap is retracted upward with the help of a blunt dissection. The flap is reflected till both the mandible and the maxilla are exposed. As a major part of the incision line lies underneath the chin and behind the ear, it is not properly visible from the frontal side.^26^

### Jaw Resection Method

The major limitation of the incision method is the limited access to the dentition owing to the anatomy of the oral cavity. Hence, if the situation permits, resection of the jaws should be performed to provide free access to the dentition**.** In some cases where the human remains are badly decomposed, burned, or fragmented, jaw resection is necessary for the examination. However, the technique must be performed meticulously without causing damage to the dentition or the face.

#### Stryker Saw Method

**Figure 9:**
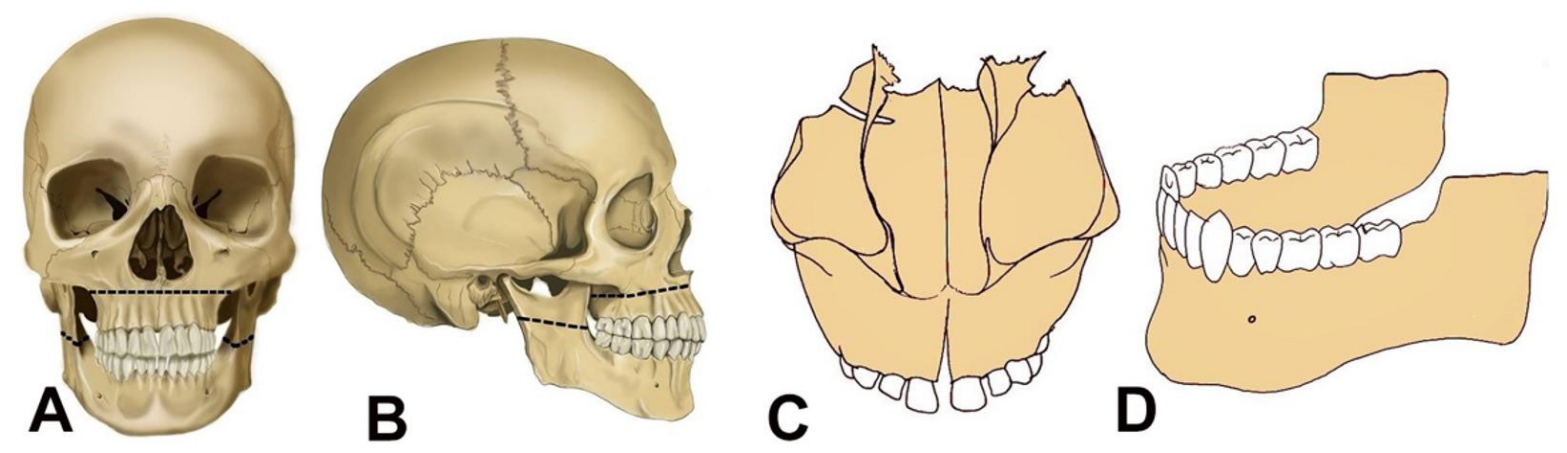
A - Stryker Saw Jaw resection method; black line represents the Osteotomy line. A – frontal view; B – lateral view; C – resected Maxilla Maxilla; B – resected mandible.

The soft tissues and muscle attachments on the lateral aspect of the mandible are first dissected. The incision is then extended through the mucobuccal fold to the lower border of the mandible from the angle of the mandible to the midline. Next, the incision is continued laterally to cut the lingual attachments, and the internal pterygoid attachments on the medial aspect of the rami are included. Furthermore, the masseter attachments on the lateral aspect of the rami are included. Once the mandible is free of all its muscle attachments, it is removed. Using a Stryker saw, an osteotomy cut is made high on the ramus, avoiding the possibly impacted third molars. After the removal of the mandible, the maxilla can be resected. The facial attachments are incised high on the malar processes superior to the anterior nasal spine. Then, using the Stryker saw, an osteotomy cut is made high on the malar process above the anterior nasal spine, avoiding the apices of the maxillary teeth. A proper-sized surgical chisel is inserted into the osteotomy cuts and firmly twisted to ensure complete bony separation. After the complete bilateral separation of the maxilla, any remaining soft tissue attachment is dissected bilaterally to remove the maxilla (Figure 9A-D).^27^

#### Chisel Mallet Method

**Figure 10:**
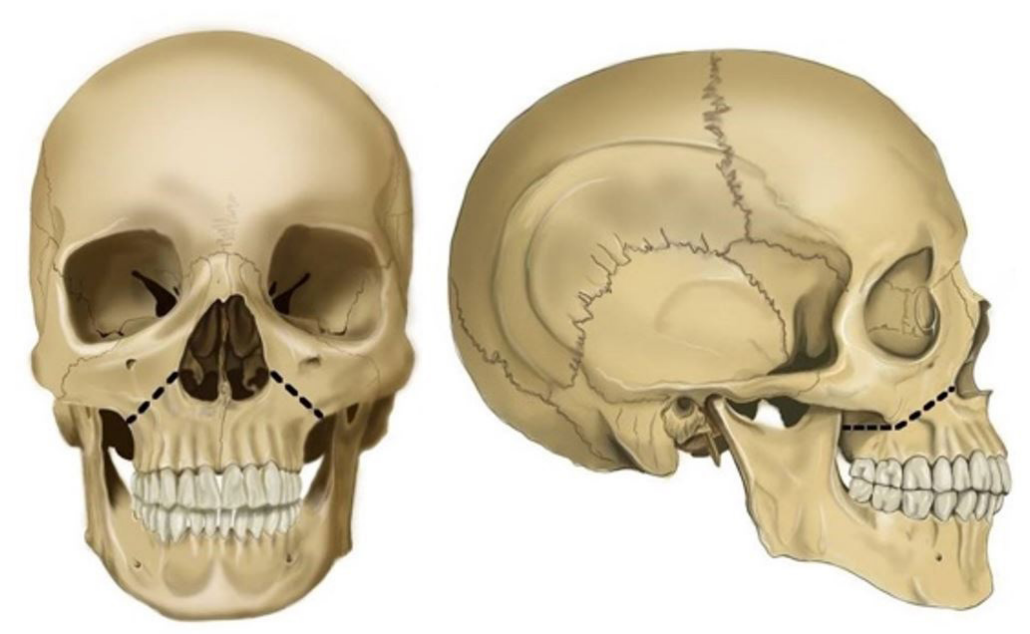
Chisel and Mallet Jaw resection method; black line represents the Osteotomy line. A – frontal view; B – lateral view.

Here, a chisel and mallet are employed to fracture the maxilla for resection. The mandible is then disarticulated from the glenoid fossae and removed. First, the lips are parted, and bilateral incisions are made along the buccal vestibule of the maxillae. All the facial attachments are incised high on the malar processes and superior to the anterior nasal spine. Subsequently, an artificial fracture is induced bilaterally using a surgical chisel and mallet**.** The fracture line should run from the nasal septum to the lateral pyriform rims. The line should travel horizontally above the maxillary teeth apices, crossing below the zygomaticomaxillary junction and traversing the pterygomaxillary junction to terminate at the pterygoid plates. This “Le Fort I” fracture enables complete removal of the maxillary bone, including all the teeth (Figure 10).

It is virtually impossible to induce a fracture on the ramus of the mandible.^29^ Thus, the mandible is exposed by dissecting all the muscular attachments, which is similar to the Stryker saw method. Once the mandible is free of its attachments, it is disarticulated form the temporomandibular joint. However, this method is highly contraindicated in case of pre-existing fractures of the facial bones. The resection fractures might be confused with any ante-mortem fractures, leading to misinterpretation of the findings.^28^

#### Pruning Shears Method

**Figure 11:**
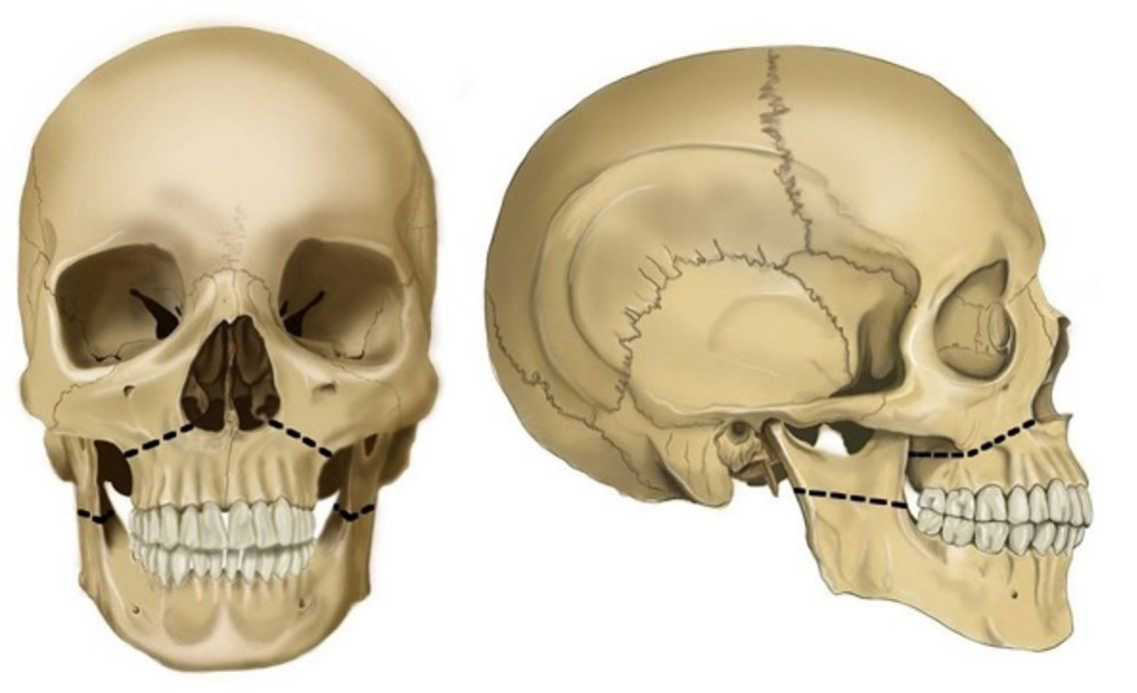
Pruning Shears Jaw resection method; black line represents the Osteotomy line. A – frontal view; B – lateral view.

A large pruning shear is used; the maxilla is first resected, and the mandible is then removed. The muscular attachments of the maxillae are incised high on the malar processes superior to the anterior nasal spine. The small blade of the pruning shears is placed within the nares and forced back into the maxillary sinus. Subsequently, a cut is made bilaterally along a plane superior to the apices of the maxillary teeth. This method ensures complete bony detachment and also incises any remaining soft tissue attachment. In case of the mandible, the muscular and soft tissue attachments are removed to permit its exposure. The blade of the shears is placed high on the lingual aspect of the ramus, near the coronoid notch, bilaterally to ensure complete separation of the bone and any loose soft tissue (Figure 11, 9C, D). This method is highly preferred for extremely brittle specimens and in case of concerns regarding the effects of vibration from the Stryker saw or the chisel mallet.^29^

#### Archimedes Screw Method

**Figure 12:**
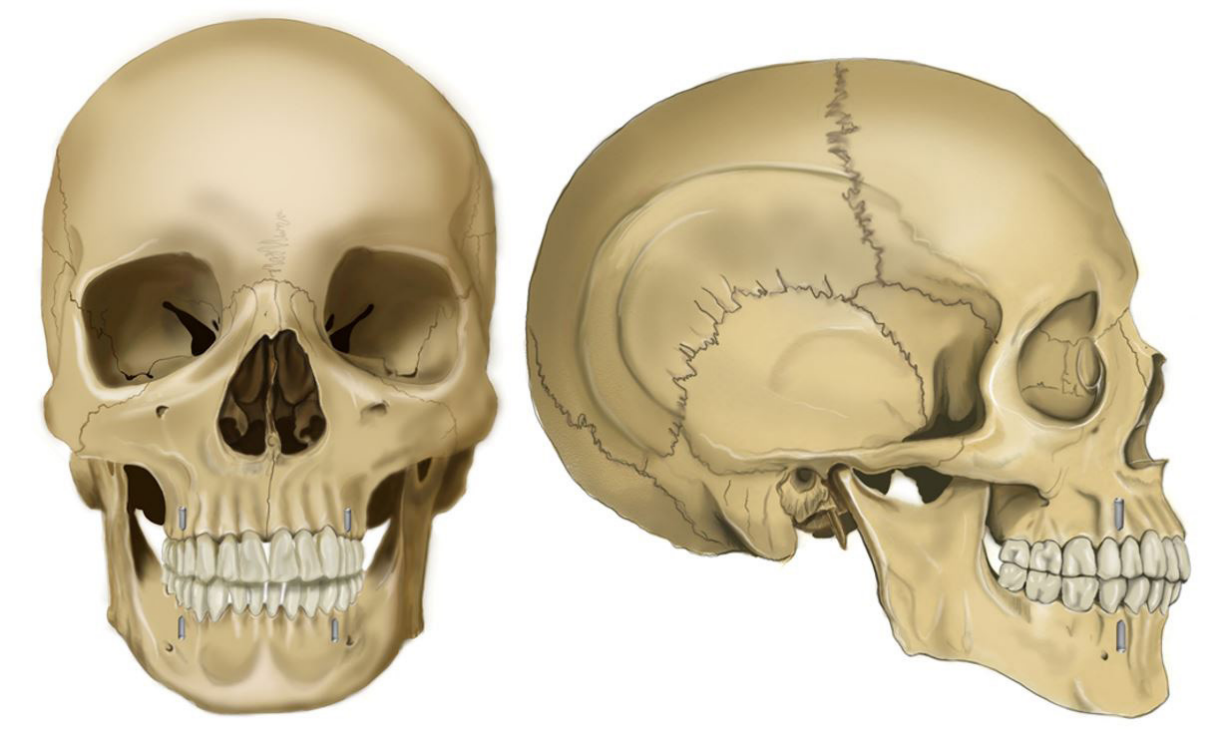
Archimedes Screw Jaw resection method, A – frontal view; B – lateral view.

This method is very useful in subjects with rigor mortis and limited mouth opening. No incision is required in this technique, and the jaws are not properly resected. The device has two screws that are attached to a central turning device. With each turn, the distance between the screws increases. Each screw is drilled into the alveolar bone between the first and second premolars bilaterally on both the jaws. The central turning device is turned to enhance the distance between the screws that separate the jaws (Figure 12). The turning process can be continued until the condylar head is dislocated from the glenoid fossae. This method does not provide good visibility, but it maintains the anatomic integrity of the facial features. The other advantage of this technique is the easy postmortem reconstruction of the face.^30^

## VIRTUAL AUTOPSY

Considering the limitations of the conventional autopsy techniques, an alternative method that is more reliable and efficient was formulated**.** In 2013, a virtual alternative was introduced by the Institute of Forensic Medicine, University of Bern, Switzerland. The virtual autopsy (Virtopsy) is a scalpel-free method that utilizes a combination of three-dimensional body surface scanning and volumetric imaging tools.^31^^,^ 32 This novel method is useful in cases of mass disasters, incinerated or charred bodies, death of high-profile individuals, bodies which are in a state of advanced putrefaction, etc. Virtopsy maintains the anatomical continuity of the body and all its organs, no matter how many times it is performed.^33^^,^ 34 With the advent of newer and more efficient technologies, the focus has been shifted to 3D surface documentation, multidetector or multi-slice computed tomography, magnetic resonance spectroscopy, image‑guided percutaneous biopsy, and postmortem angiography.^35^ The invention of the “Virtobot” is considered to be a breakthrough in the field of postmortem imaging as it is capable of conducting three-dimensional surface scans as well as postmortem image-guided soft tissue biopsies.^36^^,^ 37 The international humanitarian law states that human rights are applicable not only to the living but also to the dead.^36^ A major limitation of conventional postmortem examination is the mutilation of the body, which is considered taboo in many cultures.^38^

It is recommended to perform non-invasive procedures with skillful postmortem reconstruction of the face to make the body viewable whenever possible**.** Dental identification in such cases helps the families and the community of the dead to restore personal and social well-being.^39^ If the jaws are resected, proper placement of gauze piece or filler material is mandatory.^40^ However, dental autopsy needs to be done wherever required, either by the conventional or the virtual route. Understanding various oral incisions and resection procedures will ultimately benefit the forensic odontology community and will ease the investigation and identification procedures. With the arrival of new digital methods and enhanced awareness on forensic odontology among the judicial and law enforcement officers, acceptance of dental autopsy is likely to increase.

## CONCLUSION

The properties of the tooth make it ideal to gather postmortem data for human identification. To obtain proper access to the oral cavity of the deceased, a sound knowledge of dental autopsy techniques is crucial. A systematic and precise method needs to be adopted to aid medico-legal investigations, which makes dental autopsy an integral part of forensic investigations. With the introduction of newer, less-invasive autopsy procedures and the skillful amalgamation of conventional and modern digital techniques, the feasibility and accuracy of dental autopsy are bound to increase.
